# Supplementary material for: On the variability of the Bering Sea Cold Pool and implications for the biophysical environment
Source: PLoS One. 2022 Apr 4;17(4):e0266180. doi: 10.1371/journal.pone.0266180 (PMC8979450; doi:10.1371/journal.pone.0266180)
Supplement: Attachment — Submitted filename: ResponseToReviewer1.docx [file pone.0266180.s001.docx]

Dear Reviewer,

Thank you for providing helpful and insightful comments on our manuscript. We greatly appreciate your taking time to improve this work. We have provided our responses below in *italics*.

Reviewer #1: The authors use the Regional Arctic System Model to conduct a longterm model hindcast to describe the spatial extent and interannual variability of the Bering Sea bottom cold pool. This model has been validated through a number of studies and I find the hindcast results presented here convincing. Overall, the manuscript is relatively short and seems incomplete. The introduction is extremely limited, particularly in regard to the description of the physical oceanography in the Bering Sea, which is surprising given the topic of the manuscript. There is also highly relevant literature missing. The model results are also limited to primarily an analysis of interannual variability in the spatial extent of the bottom cold pool. I’ve expanded on these details below.

The introduction needs to be significantly expanded (there are a total of 4 sentences describing the dominate physics of the Bering Sea). The authors should provide a description of what sets up the cold pool, timing for the seasonal formation and contraction and the connection to sea ice, the interannual variability in temperature and the warm vs. cold stanzas, the relationship of the winds to sea ice extent, and the recent conditions. The authors are also missing the Kearney et al., 2020 manuscript (doi: 10.5194/gmd-13-597-2020), which is highly relevant since it is very similar to the analysis presented here (i.e. longterm model hindcast of bottom cold pool).

*We have greatly expanded the introduction, as you have suggested.*

There are a few relevant details also missing from the model methods and description section. While the authors do not need to fully recount the previous model validation studies, they should provide some brief information on the conclusions of those studies (e.g. where there any underlying biases? Which variables displayed the best performance?).

*We have added more details on the model and previous publications on model results.*

Furthermore, some of the presented figures use total area as the metric (e.g. Figure 1 and 4) but there’s no description of the boundaries that the authors use to encompass this area. The authors should either describe this in the text, or perhaps a better option is to draw out the bounding box in one of the model spatial figures.

*Thank you for pointing this out. We have added a figure (Fig. 1) that shows the area for CP calculation and geographic place names. We have also included the following text in the Results section: The area for calculation of the CP extends from 55-66^o^N latitude and across all longitudes of the Bering Sea; deep water (> 250 m) is excluded.*

Because these are model results, the authors could greatly expand the physical science analysis of the variability in the bottom cold pool. The present analysis is limited almost entirely to the interannual variability. To demonstrate the difference between high sea ice and low sea ice conditions, the authors utilize just a single year (1980 vs. 2018). Why not define a metric to quantify a low vs. high sea ice year (such as +- 1-2 std from the mean) and use this to perform a more robust analysis of the system response? The large sample size and fine temporal-spatial is a huge advantage of the long model hindcast run, which the authors are not fully utilizing.

*We have taken your suggestion and revised Fig. 2 to show the annual cycle of CP area for all of the years (1980-2018), the mean annual cycle, and +/- 1 and 2 standard deviations. This showed us that we should be comparing 2012 and 2018, since these years are outside 2 standard deviations for July CP area.*

*We also did a similar analysis for sea ice area with a revised Fig. 7. This shows that, again, years 2012 and 2018 are outside 2 standard deviations for March sea ice area. But we also see that 2001 is < -2 standard deviations, as well.*

*The findings from the statistical analysis caused us to revise Figs. 3, 4, 7, 8, 9, 10 & 11 to include more appropriate years and better definitions of the system response to extremes. While the revisions did not change any of the major conclusions, the defining of metrics and increased use of statistics greatly improved the analysis.*

The 6-month forecasts downscaled from the CFS 9-month forecasts seem very out of place in this manuscript, as there is insufficient analysis and description to conclude how skillful these forecasts are. The only skill analysis provided is a comparison to the previous year based on a spatial map of April sea ice extent. This is far too little information to gain any sense of how well the model is forecasting sea ice extent, let alone the bottom cold pool. CFS has forecasts archived stretching back decades, so the authors could do a full assessment of model forecast skill by reforecasting these previous years and comparing to the satellite product and the hindcast described in this manuscript. I suspect that this would be far beyond the scope of this current project though.

*We agree that the previous analysis was lacking on the RASM forecasts. Therefore, we have included a more robust assessment of the model’s skill at predicting the sea ice conditions and believe it is an important step to present these results for the benefit of the science community and stakeholders in the Bering Sea region.*

Minor comments:

Line 54: Remove “on the other hand”

*removed*

I recommend adding titles to each individual figure so that it’s immediately clear what is being shown without having to read through each figure caption. Especially for multi-panel plots of different years, such as Figure 5.

*Each figure has a title in bold.*

It may be a result of the upload process, but please check the resolution for all of the figures, since some of them are really low resolution (e.g. Figures 9 and 10).

*Apologies for the low-resolution figures. We have figured out the problem and have corrected it on our end. In case the problem was a result of the upload process, please note that each figure can be downloaded (in high resolution) by clicking the blue link in the upper right (e.g.* Click here to access/download;Figure;Fig9.tif)*. This was not obvious to us and we had to ask PLOS ONE for help.*
